# Downregulation of the Mitochondrial Phosphatase PTPMT1 Is Sufficient to Promote Cancer Cell Death

**DOI:** 10.1371/journal.pone.0053803

**Published:** 2013-01-10

**Authors:** Natalie M. Niemi, Nathan J. Lanning, Laura M. Westrate, Jeffrey P. MacKeigan

**Affiliations:** 1 Van Andel Research Institute, Departments of Systems Biology, Grand Rapids, Michigan, United States of America; 2 Van Andel Institute Graduate School, Grand Rapids, Michigan, United States of America; Roswell Park Cancer Institute, United States of America

## Abstract

Protein Tyrosine Phosphatase localized to the Mitochondrion 1 (PTPMT1) is a dual specificity phosphatase exclusively localized to the mitochondria, and has recently been shown to be a critical component in the cardiolipin biosynthetic pathway. The downregulation of PTPMT1 in pancreatic beta cells has been shown to increase cellular ATP levels and insulin production, however, the generalized role of PTPMT1 in cancer cells has not been characterized. Here we report that downregulation of PTPMT1 activity is sufficient to induce apoptosis of cancer cells. Additionally, the silencing of PTPMT1 decreases cardiolipin levels in cancer cells, while selectively increasing ATP levels in glycolytic media. Additionally, sublethal downregulation of PTPMT1 synergizes with low doses of paclitaxel to promote cancer cell death. Our data suggest that inhibition of PTPMT1 causes a metabolic crisis in cancer cells that induces cell death, and may be a mechanism by which cancer cells can be sensitized to currently available therapies.

## Introduction

Mitochondria, most commonly known as the ‘powerhouse of the cell,’ contain proteins with extensive post-translational modifications, including phosphorylation and acetylation. These modifications in turn influence the metabolic capacity, dynamics, and overall homeostasis of the organelle [1], [2], [3], [4]. The localization of numerous kinases and phosphatases within the mitochondria suggests that phosphorylation is an actively regulated process that plays a significant role in mitochondrial protein function [5], [6]. Despite a broad catalogue of phosphorylation events, as well as enzymes that may catalyze these events, the overall regulation of mitochondrial processes by phosphorylation, and how these events influence cellular fate, remains obscure.

PTPMT1 is a dual specificity phosphatase localized specifically and exclusively to the mitochondria [7]. It is anchored within the inner mitochondrial membrane with its phosphatase domain exposed to the matrix, placing it proximal to numerous enzymes responsible for energy production and metabolism. Interestingly, however, initial *in vitro* studies using recombinant PTPMT1 indicated that this enzyme has a clear preference for lipid substrates over protein substrates [8], suggesting that PTPMT1 could directly influence the lipid compartment of the mitochondrion. A recent study confirmed this, demonstrating that PTPMT1 functions as the mammalian phosphatidylglycerol phosphate (PGP) lipid phosphatase, catalyzing the penultimate step of the cardiolipin biosynthetic pathway [9]. Importantly, cardiolipin is synthesized and utilized exclusively within the mitochondrion, and the other critical synthetic enzymes of this pathway are known to be anchored in the inner mitochondrial membrane [10]. This places PTPMT1 specifically and selectively at the location of cardiolipin biosynthesis, and suggests that modulation of this lipid could be a critical function of this phosphatase.

Perturbations in cardiolipin homeostasis have previously been linked to apoptosis. Cardiolipin within the inner mitochondrial membrane has been shown to bind to cytochrome c, and it has been proposed that the oxidation of this lipid is required for full cytochrome c release and subsequent mitochondrial-dependent apoptosis [11]. Additionally, cardiolipin has been implicated in the targeting of numerous pro-apoptotic proteins to the mitochondria, including tBID, a BH3-only protein known to induce cytochrome c release through the promotion of mitochondrial outer membrane permeabilization [12]. As a block in apoptosis is considered to be a hallmark of cancer [13], dysregulation of cardiolipin could affect the tumorigenic potential of cells by influencing their ability to undergo cell death. Additionally, alterations in the cardiolipin biosynthetic pathway have also been linked to apoptosis. RNAi-mediated knockdown of cardiolipin synthase (CLS1; gene name *CRLS1*) is sufficient to release cytochrome c from the inner mitochondrial membrane, and sensitizes cancer cells to apoptotic stimuli [14].

Despite striking homology between PTPMT1 and another lipid phosphatase, PTEN, within their catalytic domains [8], a chemical screening approach identified a compound, alexidine dihydrochloride, that is specific for PTPMT1 *in vitro* [15]. Alexidine dihydrochloride is a bisbiguanide compound initially identified for its anti-bacterial properties, but interestingly has also identified as an inducer of mitochondrial dysfunction and apoptosis [16]. As PTPMT1 is localized specifically within the mitochondria, alexidine dihydrochloride could be modulating mitochondrial dysfunction and subsequent pro-apoptotic affects through the inhibition of PTPMT1 phosphatase activity.

The proposed role of cardiolipin in the normal apoptotic process, coupled with the robust cell-death inducing phenotype of alexidine dihydrochloride, led us to hypothesize that downregulation of PTPMT1 gene expression would induce an apoptotic cellular fate in cancer cells. To explore this hypothesis, we used RNAi to knockdown PTPMT1 and determine the cellular consequence of loss of this specific gene product. Additionally, we explored the effects of the PTPMT1-targeting compound, alexidine dihydrochloride, in its ability to both induce cell death in cancer cells as well as to sensitize these cells to chemotherapeutics at sublethal doses. Here, we demonstrate that RNAi-mediated silencing of PTPMT1 is sufficient to induce cancer cell death. This cell death phenotype is coupled with striking metabolic changes, including a significant downregulation of cardiolipin upon loss of PTPMT1, as well as an increase in ATP levels selectively in glucose-containing media. These data illuminate a role for PTPMT1 as a critical survival gene in cancer cells, and suggest that targeting this phosphatase could be an effective way to sensitize cancer cells to currently available chemotherapeutics.

## Results

### RNAi-mediated Knockdown of PTPMT1 Causes Cell Death

**Figure 1:**
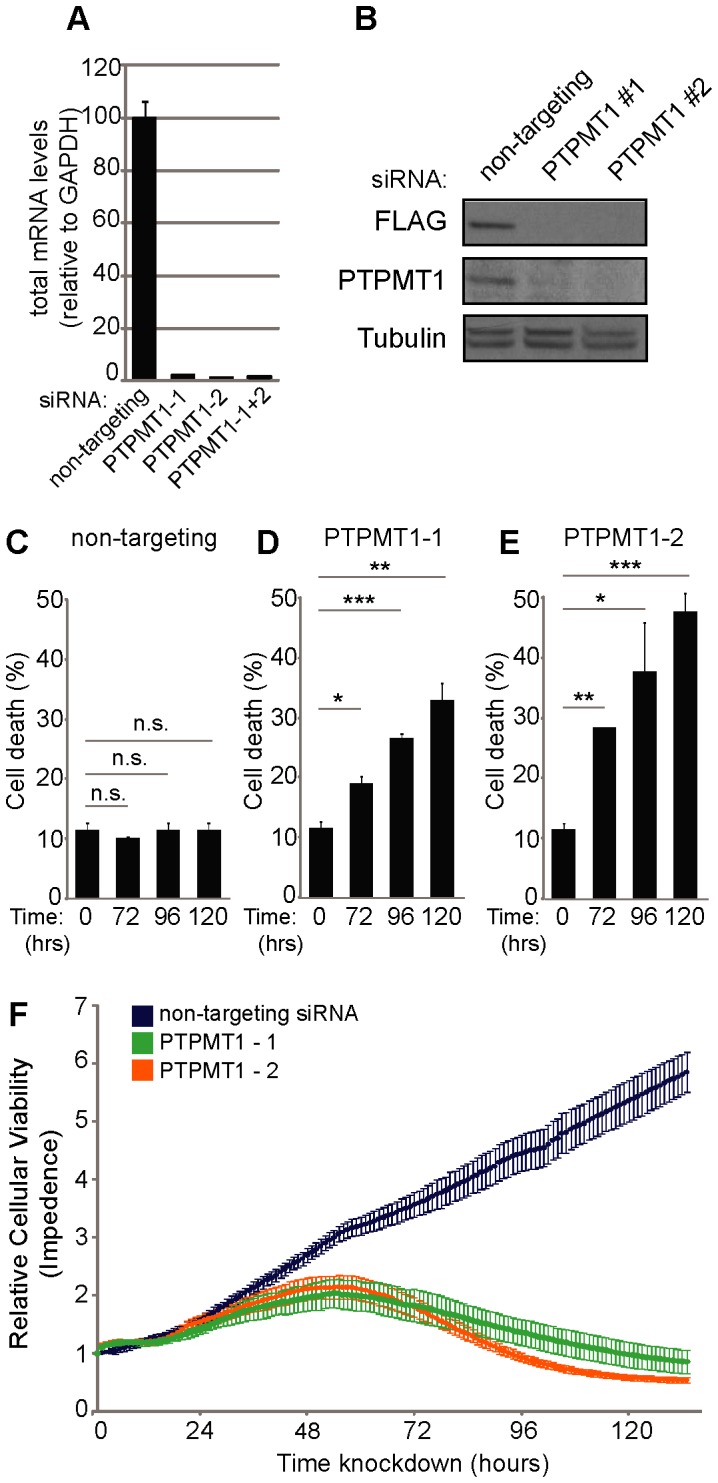
RNAi-mediated downregulation of PTPMT1 induces cell death in HeLa cells. (A) Quantitative RT-PCR analysis of PTPMT1 mRNA levels in HeLa cells after knockdown with two non-overlapping PTPMT1-specfic siRNAs or a non-targeting control. (B) HeLa cells were transfected with two PTPMT1 siRNAs or a non-targeting control for 48 hr. In one experiment, PTPMT1 was overexpressed (with a FLAG-tag) for 20 hr (total siRNA transfection time 48 hr) and expression was probed with a FLAG antibody (top panel). siRNA knock down was also determined for endogenous PTPMT1 (middle panel). Tubulin (bottom panel) demonstrates equal loading of protein lysates in all lanes. (C–E) HeLa cells were transfected with control or PTPMT1 siRNAs and collected after indicated times, at which cell death was assayed by propidium iodide exclusion. Error bars indicate standard deviation of three experiments. Statistical significance was calculated using a student’s t test; * - p<0.05; ** - p<0.01; *** - p<0.001. (F) HeLa cells were transfected with control or PTPMT1 siRNAs and assayed for viability in real time (>120 hours) using the xCELLigence system.

To examine the effects of knocking down PTPMT1 on cell fate, we utilized two unique siRNA sequences targeting non-overlapping regions of the PTPMT1 open reading frame. Each of these siRNAs provides robust knockdown of the PTPMT1 mRNA transcript 30 hours post transfection, with both PTPMT1 siRNAs giving over 98% knockdown relative to GAPDH (Figure 1A). To determine if these siRNAs effectively disrupt PTPMT1 protein expression, we determined the ability of each siRNA to knock down overexpressed, FLAG-tagged protein (Figure 1B, top panel) as well as endogenous PTPMT1 (Figure 1B, middle panel). These experiments demonstrate clear silencing of PTPMT1 protein expression 48 hours after transfection with each siRNA.

Interestingly, both of these siRNA sequences cause cell death after 72 hours of knockdown, which became more prominent at 96 and 120 hours post-knockdown (Figures 1C–E, F). To determine the percentage of cells undergoing cell death, we performed FACS analysis of propidium iodide positive cells at 72, 96, and 120 hours post-siRNA knockdown in cells transfected with either PTPMT1 siRNA or a non-targeting control. As expected, the cells transfected with a non-targeting control siRNA showed little cell death over the 120 hour period monitored, with 10.8% maximal cell death seen during this time frame (which is only slightly higher than basally treated HeLa cells (p = 0.2–0.9, n.s.)). On the contrary, either siRNA targeting PTPMT1 caused robust cell death, particularly evident at 96 hours (PTPMT1 siRNA#1 p<0.001; PTPMT1 siRNA#2 p<0.05) and 120 hours (PTPMT1 siRNA#1 p<0.01; PTPMT1 siRNA#2 p<0.001), which proved significant over both untreated cells (0 hr condition, Figures 1C–E) and time-matched non-targeting controls.

To determine more precise kinetics of this cell death, we monitored real-time cellular viability upon transfection of HeLa cells with either a non-targeting control siRNA (blue) or one of the two unique PTPMT1 siRNA sequences (PTPMT1-1, green; PTPMT1-2, orange) (Figure 1F). Recapitulating the propidium iodide cell death data, both siRNA sequences began to slow cellular proliferation approximately 72 hours post-transfection and promoted significant cellular detachment (indicative of cell death) at 96 and 120 hours post-transfection. These data demonstrate that expression of PTPMT1 is required for cellular viability in HeLa cells, and suggests that PTPMT1 may be required for viability in cancer cells.

### PTPMT1 Knockdown Causes Bax/Bak Dependent Apoptosis

**Figure 2:**
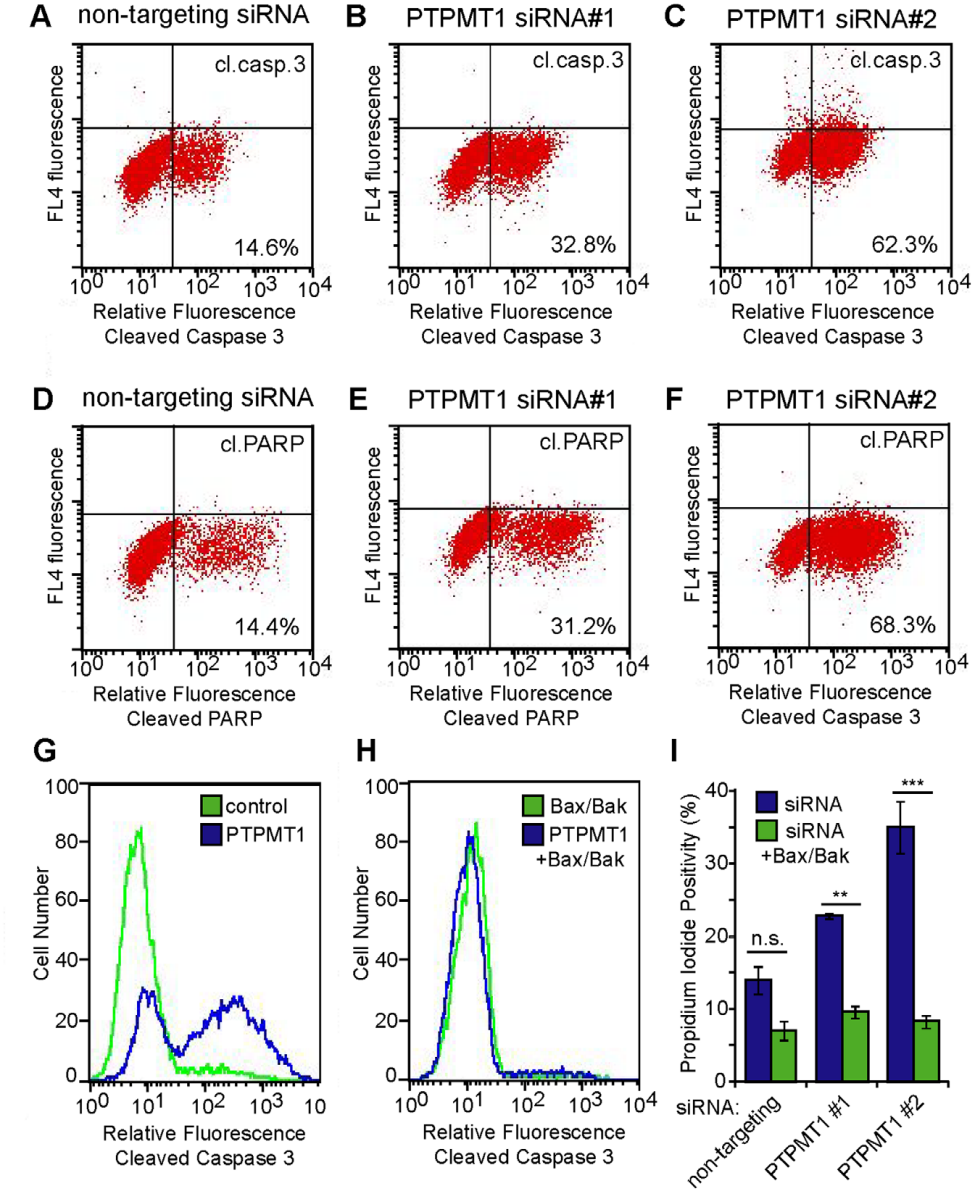
PTPMT1 knockdown induces mitochondrial-dependent apoptosis. (A–F) HeLa cells were transfected with control non-targeting siRNA (A, D), PTPMT1 siRNA#1 (B, E), or PTPMT1 siRNA#2 (C, F) for 96 hours. Cells were collected and stained for cleaved caspase-3 (top panels) or cleaved PARP (bottom panels) under each condition, with positivity indicated as an increase in fluorescence on the FACS histogram (lower right quadrant). (G–I) HeLa cells were transfected with control, PTPMT1, and/or BAX/BAK siRNAs for 96 hours. Cells were collected and stained for cleaved caspase-3 (G, H) or propidium iodide positivity (I). Error bars indicate standard deviation of three experiments. Statistical significance was calculated using a student’s t test; * - p<0.05; ** - p<0.01; *** - p<0.001.

Previous reports have associated downregulation of cardiolipin levels with cytochrome c release and sensitization to apoptotic cell death in cancer cells as well as cardiomyocytes [14]; [17]. Thus, we hypothesized that cell death phenotype seen in response to PTPMT1 knockdown was an intrinsic, or mitochondrial-dependent, apoptotic response. To determine whether PTPMT1 knockdown induces apoptosis, we assayed caspase-3 cleavage, as well as the cleavage of its well characterized substrate, PARP. Figures 2A and B demonstrate that at 96 hours post-transfection, cells transfected with a non-targeting siRNA do not show enrichment in either caspase-3 or PARP cleavage. However, transfection with either PTPMT1-targeting siRNA induced a robust enrichment in both caspase-3 (Figures 2C and D) and PARP cleavage (Figures 2E and F). Importantly, both caspase-3 cleavage and PARP cleavage track closely together in these experiments, emphasizing the apoptotic phenotype after PTPMT1 knockdown.

While caspase-3 is a marker of apoptotic cell death, it is activated in response to both intrinsic and extrinsic apoptotic cues. To determine whether PTPMT1 knockdown induced mitochondrial-dependent apoptosis, we used siRNAs to dually knockdown the pro-apoptotic proteins BAX and BAK, whose expression is required for classical, mitochondrial-dependent apoptosis [18]. In the presence of a non-targeting control normalized for siRNA concentration, PTPMT1 induced caspase-3 cleavage with similar kinetics as seen in Figure 2C (Figure 2G). Strikingly, however, Bax/Bak dual knockdown completely eliminated PTPMT1-mediated apoptotic induction (Figure 2H). To ensure that PTPMT1 knockdown is not causing a non-apoptotic cell death in the presence of Bax/Bak knockdown, we repeated these experiments and assayed cells for propidium iodide positivity. Consistent with our previous data, PTPMT1 siRNA causes an increase in propidium iodide positivity over a non-targeting control (Figure 2I, blue bars). This increase in cell death is completely blocked by Bax/Bak knockdown, which provides significant protection from propidium iodide uptake seen in the PTPMT1 knockdown cells (Figure 2I, green bars; p<0.01 for PTPMT1 siRNA#1, p<0.001 for PTPMT1 siRNA#2). These data demonstrate that Bax/Bak expression is required for PTPMT1-induced cell death, and suggest that PTPMT1 knockdown induces an intrinsic, mitochondrial-dependent apoptotic cell death that is dependent upon cytochrome c release.

### PTPMT1 Knockdown Causes Apoptotic Cell Death in Multiple Cancer Cell Lines

**Figure 3:**
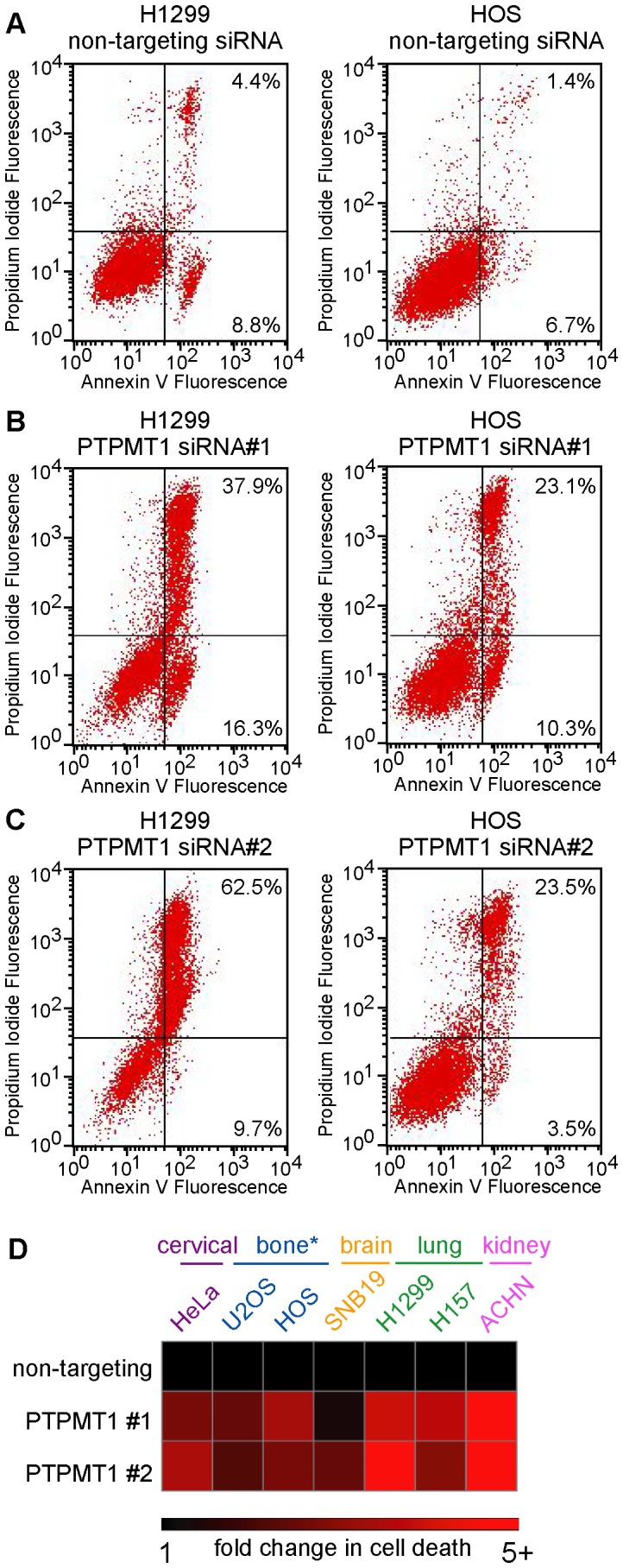
PTPMT1 knockdown induces apoptosis in many cancer cell lines. (A–C) The lung carcinoma cell line H1299 and the osteosarcoma cell line HOS were transfected with non-targeting (A), PTPMT1 siRNA#1 (B) or PTPMT1 siRNA#2 (C). After transfection of these siRNAs for 120 hours, the population of cells undergoing cell death and apoptosis was measured through propidium iodide positivity (y-axis) and Annexin V positivity (x-axis). (D) A panel of highly transfectable cell lines derived from many tissue types (see colored labels above heatmap) were transfected with non-targeting or PTPMT1 siRNAs. Cell death and apoptosis were assayed by propidium iodide and Annexin V positivity as in (A–C). Fold change in apoptosis was determined by normalizing percent cell death seen in cells transfected with a non-targeting control to 1, with fold change reflecting the amount of cell death above this threshold in PTPMT1 knockdown cells. The heatmap shows a range of fold change from 1 (black squares) to 5+ (five or greater fold, red squared).

While our data has shown that two independent, non-overlapping PTPMT1-targeted siRNAs promote an apoptotic cell death in HeLa cells, gene inactivation of PTPMT1 in mouse embryonic fibroblasts or hematopoietic cells has little effect on cellular viability. We hypothesized that downregulation in transformed cells could have an alternate effect on cell fate relative to these non-transformed, primary cells. To determine if the loss of PTPMT1 induces cell death in only a subpanel of cell lines (such as HeLas), or has similar effects in other transformed cells, we chose a panel of cancer cell lines that are highly amenable to siRNA transfection (all >95% transfectable, data not shown). These cell lines cover a broad range of tumor types (sarcomas and carcinomas); tissues of origin (bone, brain, lung, and kidney); and tumorigenicity *in vivo*. Similar to our earlier experiments, these cell lines were transfected with a non-targeting siRNA or PTPMT1 siRNA#1 or #2. 120 hours post-transfection, the cells were collected and analyzed for apoptosis via Annexin V positivity (y-axis Figures 3A–C) and propidium iodide positivity (x-axis, Figures 3A–C). As shown in Figures 3B and C, the transfection of both PTPMT1 siRNAs caused robust increased in Annexin V and PI staining, indicating that these siRNAs cause these cell lines to undergo apoptosis. In most cell lines tested, this was a robust response, with an average 3.8- and 3.9- fold increase in cell death occurring in 6 of 8 cell lines tested with PTPMT1 siRNA #1 or siRNA# 2, respectively (Figure 3D). We found that 786-0 cells, a renal carcinoma cell lines, were not significantly affected by PTPMT1 knockdown with either siRNA (data not shown). These data demonstrate that PTPMT1 knockdown causes cell death in this subset of cancer cell lines, suggesting its expression is critical to the survival of these cell lines.

### Titration of PTPMT1 Knockdown Alters Effects on Cellular Viability

**Figure 4:**
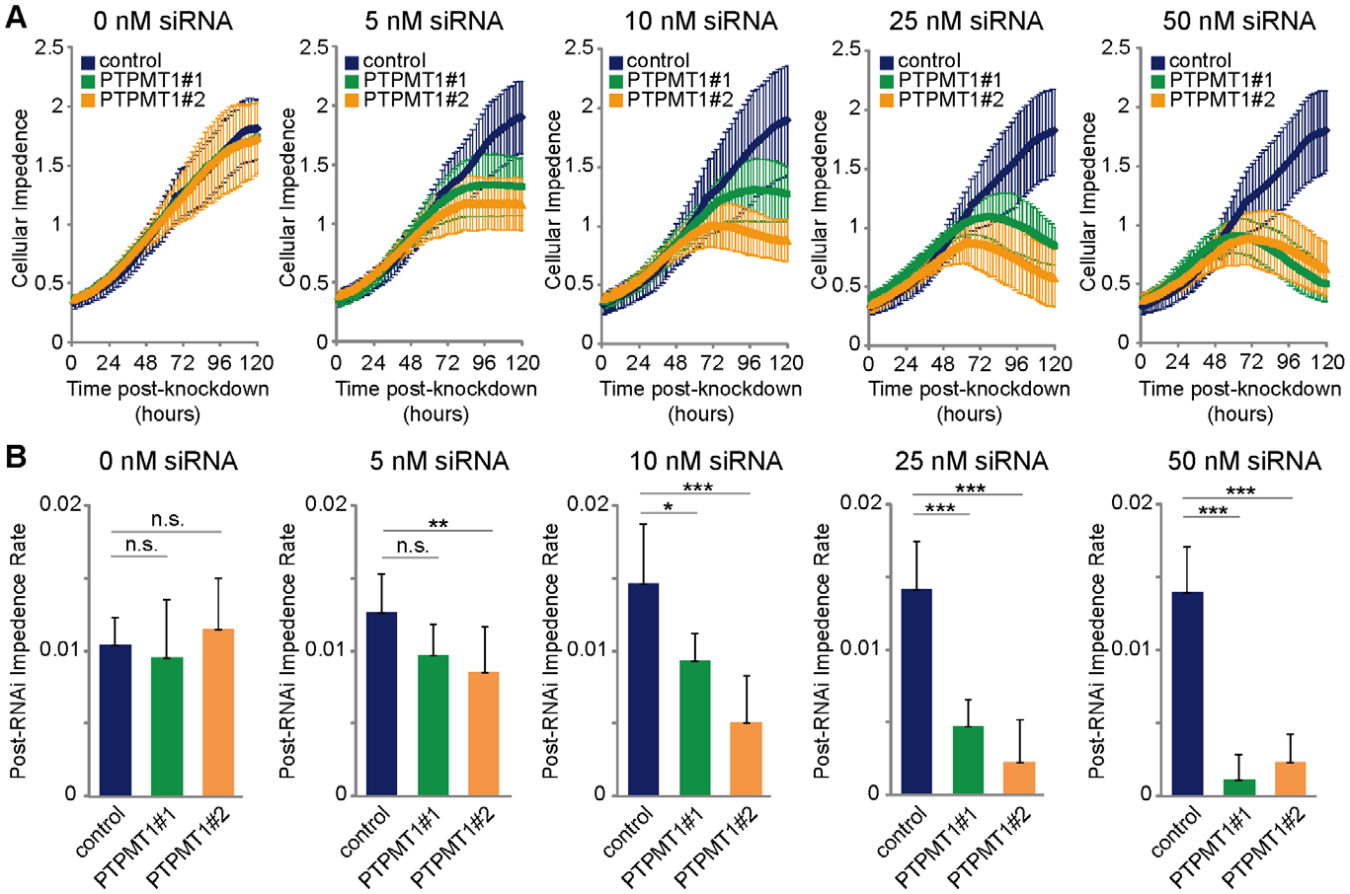
Differential PTPMT1 knockdown alters the absolute level of HeLa cell apoptosis. (A) HeLa cells were transfected with a dose response of non-targeting (blue line), PTPMT1#1 (green line) or PTPMT1#2 (orange line) siRNA over 120 hours. Cells were transfected with 0, 5, 10, 25, or 50 nM of each siRNA and real-time viability was tracked using xCELLigence. (B) Rate of change in cellular impedance, reflecting changes in proliferation (positive slope) and/or viability (negative slope) in cells transfected with control or PTPMT1 siRNAs. A lower rate of change in impedance is associated with decreased cellular attachment, indicative of cell death. Error bars indicate standard deviation of three experiments. Statistical significance was calculated using a student’s t test; * - p<0.05; ** - p<0.01; *** - p<0.001.

RNAi titration has been used previously to determine a gene expression threshold required for cellular viability [19]. In these experiments, siRNA oligonucleotides were diluted and titrated down to a low nanomolar level to understand the relative expression level that a gene must sustain to induce or prevent a specific cellular fate, such as cell death. To more fully understand the siRNA-induced cell death we had seen in PTPMT1 knockdown cells, we titrated each PTPMT1 siRNA, as well as a non-targeting siRNA control, and examined the effects of these differential knockdowns on HeLa cell viability and proliferation. As expected, titration of a non-targeting control from 50 nM down to 5 nM had little effect on the viability or proliferation capacity of HeLa cells over a 120 hour timeframe (Figure 4A, B; navy blue data points). PTPMT1 expression was differentially downregulated by titration of both unique PTPMT1 siRNAs, with increasing knockdown efficiency as each siRNA concentration increased from 5 nM to 50 nM (data not shown). Interestingly, the titration of each PTPMT1 siRNA altered the proliferative and viability profiles of PTPMT1 knockdown cells; while 25 to 50 nM of PTPMT1 siRNA #1 or #2 is sufficient to induce cellular detachment, indicative of cell death, 5 to 10 nM of this same siRNA seems to halt proliferation without inducing cellular detachment, as indicated by a neutral slope on the real time viability plots (Figure 4A, B).

### Knockdown of PTPMT1 Sensitizes Cancer Cells to Sublethal Doses of Paclitaxel

**Figure 5:**
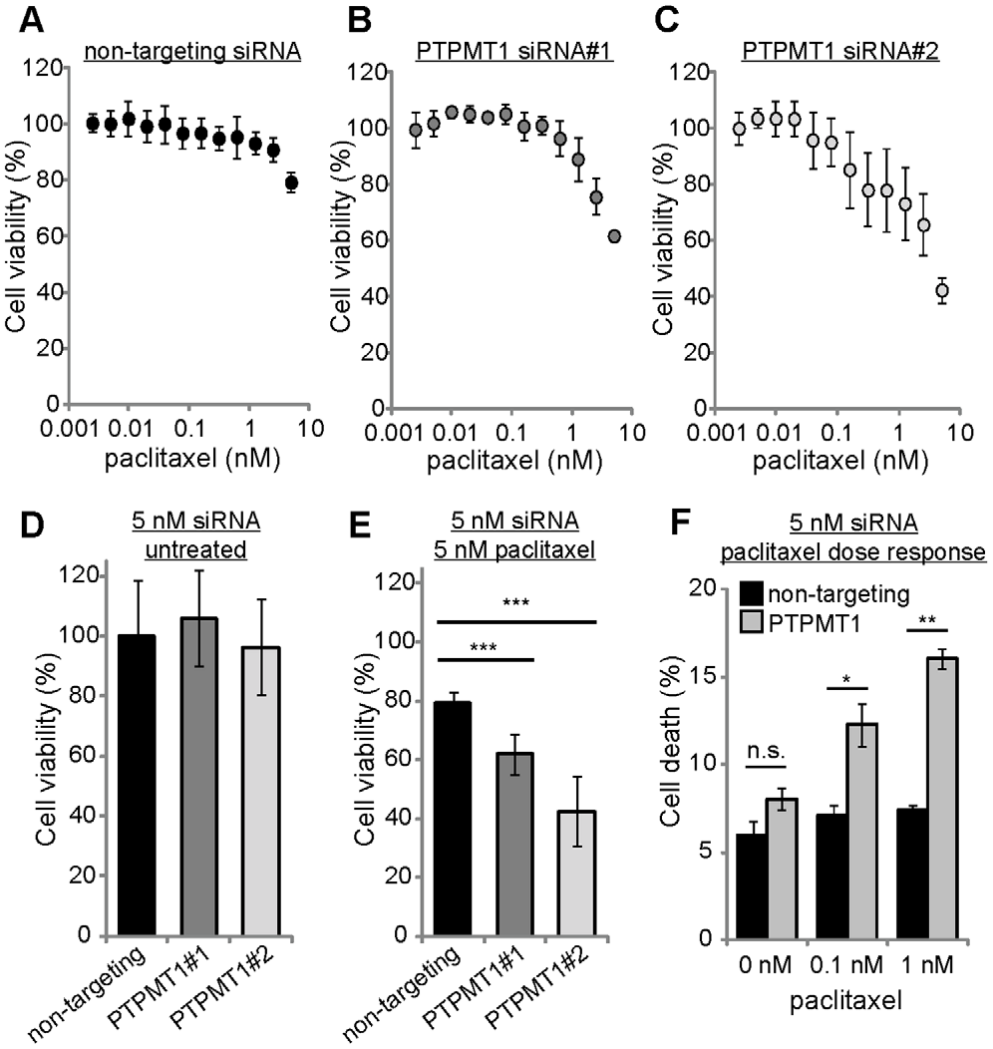
Sublethal PTPMT1 knockdown sensitizes cells to the chemotherapeutic paclitaxel. (A–C) HeLa cells were transfected with 5 nM non-targeting siRNA (A), PTPMT1 siRNA#1 (B), or PTPMT1 siRNA#2 (C) for 30 hours before treatment with a dose response of sublethal paclitaxel (up to 5 nM) for 24 hours. Viability was measured using Cell Titer Glo. (D) Viability of untreated HeLa cells transfected with each siRNA at 54 hours. (E, F) Quantification of HeLa cell viability (E) or HeLa cell death by propidium iodide exclusion (F) with 5 nM non-targeting or PTPMT1 siRNA (54 hr total treatment) and 5 nM paclitaxel treatment (24 hr total treatment). For each experiment, error bars indicate standard deviation of three experiments. Statistical significance was calculated using a student’s t test; * - p<0.05; ** - p<0.01; *** - p<0.001.

The observation that differential knockdown levels of PTPMT1 changed cancer cell fate raised the possibility that suboptimal knockdown of PTPMT1 (to a level not sufficient to induce apoptosis) may sensitize cancer cells to low levels of chemotherapeutic which would otherwise be relatively inefficient. To test this hypothesis, we transfected 5 nM of control or PTPMT1-specific siRNAs into HeLa cells for 30 hours before exposing these cells to low concentrations (5 nM) of the chemotherapeutic paclitaxel for an additional 24 hours. Figure 5 demonstrates that transfection of HeLa cells with low levels of non-targeting siRNA does not promote sensitization to low doses of paclitaxel, with 5 nM paclitaxel inducing a 21% reduction in cellular viability non-targeting siRNA (Figure 5A). Transfection of 5 nM PTPMT1 was sufficient to sensitize cells to paclitaxel treatment: while 21% of control cells underwent cell death in the presence of 5 nM paclitaxel, 38% or 57% of 5 nM PTPMT1 siRNA-treated cells underwent significantly more cell death under the same conditions (siRNAs 1 and 2, respectively; p<0.01 for PTPMT1 siRNA#1, p<0.001 for PTPMT1 siRNA#2, Figures 5B and C). Importantly, 5 nM of each siRNA targeting PTPMT1 does not cause significant cellular toxicity at 72 hours (Figure 5D, E), demonstrating that the death induced by 5 nM PTPMT1 siRNA combined with 5 nM paclitaxel treatment is likely a synergistic interaction. To confirm these observations, we determined the population of cells undergoing cell death upon paclitaxel exposure after incubation with 5 nM PTPMT1 siRNA. While neither 0.1 nM nor 1 nM paclitaxel induces propidium iodide positivity in cells transfected with a non-targeting siRNA (Figure 5F, black bars), both of these doses of paclitaxel significantly increase the population of cells undergoing cell death after transfection with PTPMT1 siRNAs (Figure 5F, grey bars). Overall, these data demonstrate that sub-lethal RNAi-mediated knockdown of PTPMT1 is sufficient to sensitize HeLa cells to sub-lethal doses of the chemotherapeutic paclitaxel.

### PTPMT1 Knockdown Induces Significant Metabolic Changes in Cancer Cells

**Figure 6:**
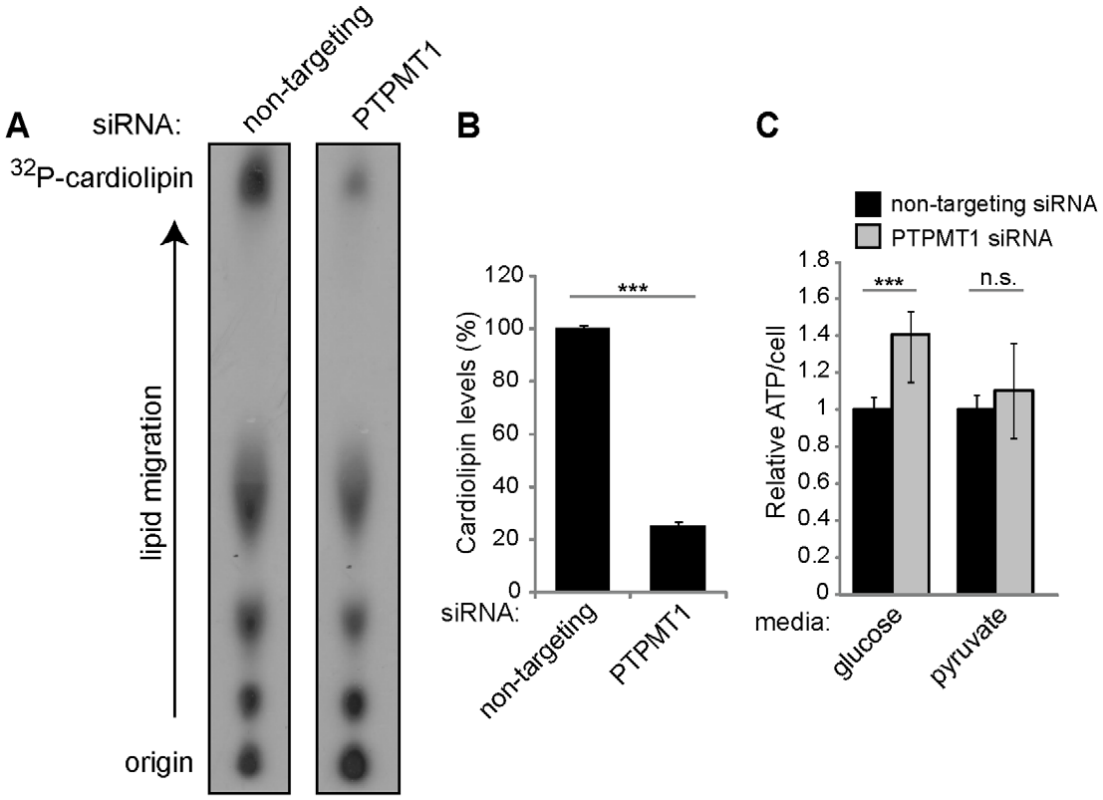
PTPMT1 knockdown alters cardiolipin levels and total ATP/cell in cancer cells. (A, B) HeLa cells were transfected with non-targeting or a pool of PTPMT1 siRNAs (#1+ #2) for 72 hours. Cells were incubated with ^32^P-orthophosphate and radiolabeled lipids were extracted and resolved via thin layer chromatography. Cardiolipin is shown as the highest spot imaged on the TLC plates (A), and total cardiolipin levels were quantified (B). (C) Total ATP/cell was assayed in cells transfected with a non-targeting or PTPMT1 pool of siRNAs for 72 hours. ATP/cell was determined in cells growing in both high glucose-containing media or media containing only pyruvate. Error bars indicate standard deviation of at least three experiments. Statistical significance was calculated using a student’s t test; * - p<0.05; ** - p<0.01; *** - p<0.001.

Upon being identified as a mitochondrial phosphatase, PTPMT1 function was linked with mitochondrial metabolism when it was shown that PTPMT1 knockdown causes a robust increase in cellular ATP levels in pancreatic beta cells [7]. Recently, PTPMT1 was identified as a key phosphatase in the biosynthesis of cardiolipin, a key mitochondrial lipid linked to both mitochondrial metabolism and apoptosis. Importantly, deficiencies in cellular cardiolipin levels have been linked to apoptosis [14], [20], leading us to investigate if PTPMT1 knockdown in cancer cells alters this mitochondrial lipid. HeLa cells were transfected with a non-targeting or pool of PTPMT1 siRNAs (siRNAs #1+ #2) for 72 hours, whereupon cellular lipids were radiolabeled, extracted, and resolved via thin layer chromatography. Consistent with previously published results [9], ablation of PTPMT1 expression causes a robust decrease in cardiolipin levels relative to cells expressing a non-targeting control (77% decrease, p<0.001, Figures 6A and B). These changes in mitochondrial lipid composition have been demonstrated to alter the metabolic capacity of cells, with the loss of PTPMT1 expression resulting in an increase in ATP levels resulting from a compensatory shift to enhanced glycolysis [9]. To determine if mitochondrial metabolism is altered in PTPMT1 knockdown cells, ATP levels were measured in cells expressing either a non-targeting or PTPMT1 siRNA pool (siRNAs #1+ #2). As shown in Figure 6C, PTPMT1 knockdown cells have significantly more ATP per cell than cells transfected with a non-targeting control when grown in standard media containing glucose (40.5% more ATP/cell, p<0.001). To determine if this increase is dependent upon glucose metabolism, we also performed this experiment in cells cultured in media containing only pyruvate as a carbon source, disallowing the glycolyic program to be engaged. In these conditions, there is no significant change in ATP levels in PTPMT1 knockdown cells relative to control cells (10% more ATP/cell, Figure 6C, p = 0.178). Collectively, these data suggest that PTPMT1 knockdown decreases cardiolipin levels in HeLa cells, leading to a transient increase in ATP levels independent of mitochondrial metabolism, most likely due to enhanced glycolysis.

### Alexidine Dihydrochloride Inhibits PTPMT1 *in vitro* and Induces Apoptosis in Cancer Cells

**Figure 7:**
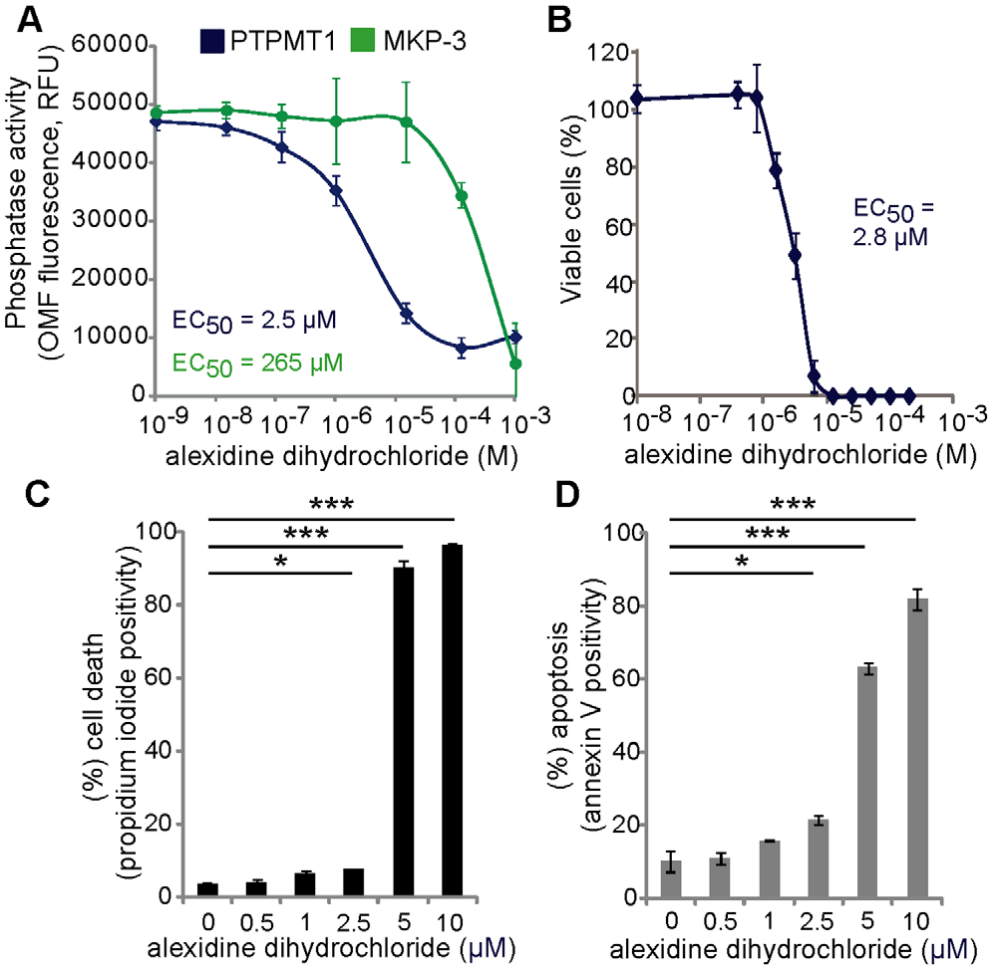
Alexidine dihydrochloride specifically inhibits PTPMT1 and induces cell death. (A) Recombinant PTPMT1 and MKP-3 were treated with various concentrations of alexidine dihydrochloride, *in vitro* phosphatase assays were performed, and the resulting effects on enzymatic activity measured. The IC_50_ for each enzyme was calculated and displayed using SigmaPlot. (B) HeLa cells were treated with a dose response of alexidine dihydrochloride for 24 hours and resulting changes in viability were measured using Cell Titer Glo. (C, D) HeLa cells were treated with alexidine dihydrochloride for 24 hours before measuring cell death (C) by propidium iodide staining (C) or induction of apoptosis by Annexin V staining (D). For each experiment, error bars indicate standard deviation of three experiments. Statistical significance was calculated using a student’s t test; * - p<0.05; ** - p<0.01; *** - p<0.001.

A recent publication has identified the compound alexidine dihydrochloride as a selective inhibitor of PTPMT1 *in vitro* [15]. We sought to validate this data using recombinant PTPMT1. As a control, we also tested the ability of alexidine dihydrochloride to inhibit MKP-3, a dual specificity phosphatase with little homology to PTPMT1. Consistent with data generated by Doughty-Shenton et al., alexidine dihydrochloride inhibits the phosphatase activity of PTPMT1 with a significantly lower IC_50_ than other phosphatases, including MKP-3 (2.5 µM for PTPMT1 v. 265 µM for MKP-3, Figure 7A). Before its identification as a specific inhibitor of PTPMT1 phosphatase activity, alexidine dihydrochloride had been shown to induce apoptosis in cancer cells [21]. To determine if alexidine dihydrochloride induced cell death in our experimental system, we performed a simple dose response experiment in which we exposed HeLa cells to two-fold serial dilutions of alexidine dihydrochloride for 24 hours. Consistent with the previous report, alexidine dihydrochloride promoted robust cell death, with an EC_50_ near 2.8 µM (Figure 6B). Importantly, this concentration is near the concentration that specifically inhibits recombinant PTPMT1 *in vitro*, suggesting that the specific targeting of this phosphatase could be responsible for this cell death phenotype.

To confirm that alexidine dihydrochloride was inducing an apoptotic cell death similar to what we saw with PTPMT1 siRNA knockdown, we exposed HeLa cells to a dose response of alexidine dihydrochloride, determining which concentrations induce cell death (via propidium iodide staining) and whether this cell death was apoptotic (by determining Annexin V positivity). These data demonstrate that there is a dramatic increase in HeLa cell death between 2.5 and 5 µM alexidine dihydrochloride treatment (Figure 7C), which agrees with our initial dose response curve. Importantly, this shift to cell death is apoptotic, as a similar increase in Annexin V positive cells is seen with the same dose response (Figure 7D), and is in the low micromolar range, which specifically targeted PTPMT1 phosphatase activity *in vitro*. These data suggest that the apoptotic phenotype induced by alexidine dihydrochloride treatment could be due to inhibition of PTPMT1 phosphatase activity.

### Sublethal Doses of Alexidine Dihydrochloride are Sufficient to Sensitize Cancer Cells to Low Levels of Paclitaxel but are Not Completely Dependent upon PTPMT1 Inhibition

**Figure 8:**
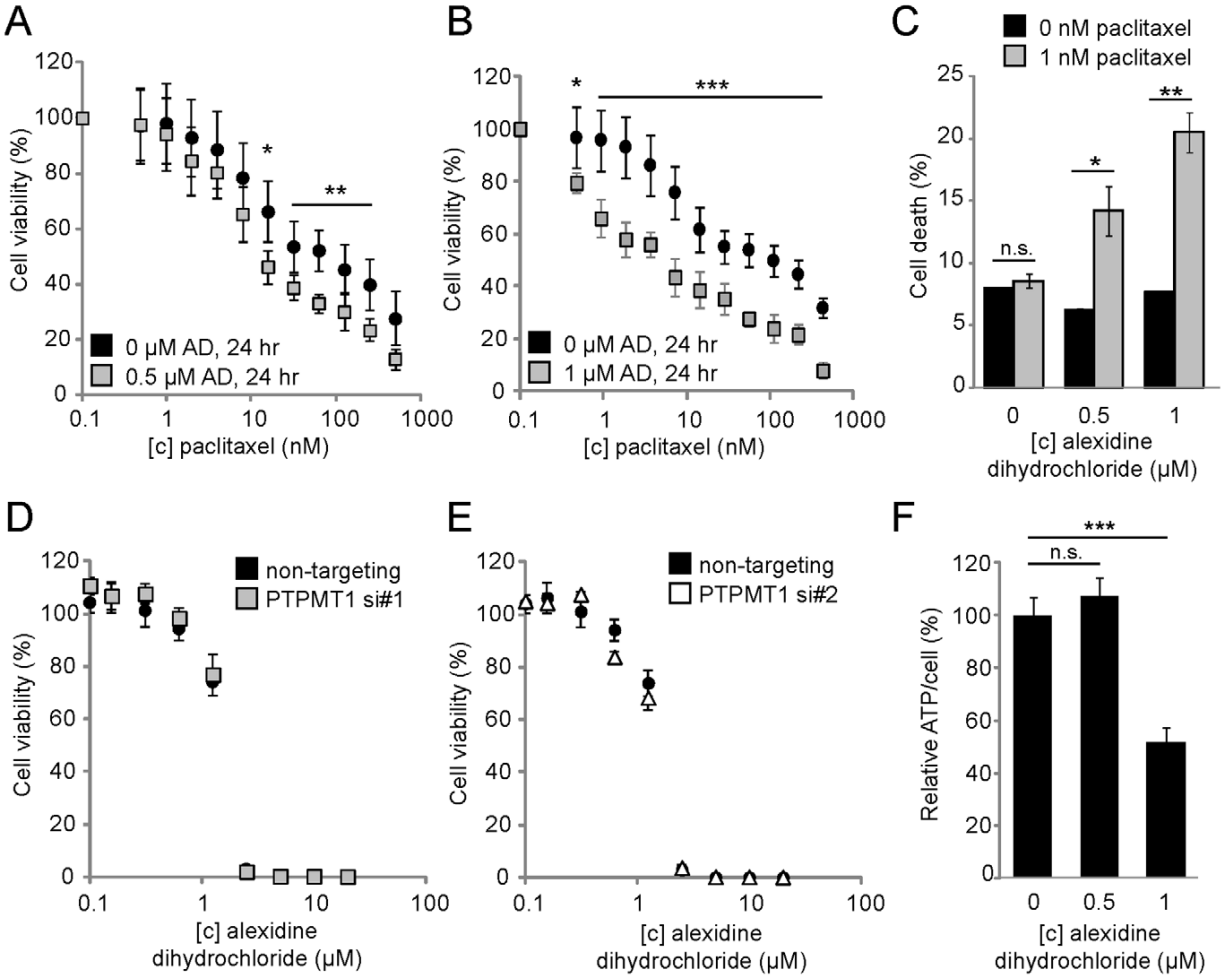
Sublethal doses of alexidine dihydrochloride are sufficient to sensitize HeLa cells to the chemotherapeutic paclitaxel but are independent of PTPMT1 expression. (A, B) HeLa cells were treated with 0.5 µM (A) or 1 µM (B) alexidine dihydrochloride, or a vehicle only control (DMSO, 0 µM condition, (A, B)) for 24 hours. Cells were then exposed to a dose response of paclitaxel for an additional 48 hours. Changes in viability were measured using Cell Titer Glo. (C) HeLa cells were treated with 0.5 µM or 1 µM alexidine dihydrochloride for 24 hrs before being exposed to 1 nM paclitaxel for an additional 24 hrs. Cell death was assayed by propidium iodide positivity. (D, E) HeLa cells were transfected with non-targeting siRNA (black circles), PTPMT1 siRNA#1 (grey squares), or PTPMT1 siRNA#2 (white triangles) for 30 hours before being exposed to a dose response of alexidine dihydrochloride for 24 hours. Viability was assayed using Cell Titer Glo. (F) Total ATP/cell was assayed in cells treated with alexidine dihydrochloride for 24 hrs. For each experiment, error bars indicate standard deviation of three experiments. Statistical significance was calculated using a student’s t test; * - p<0.05; ** - p<0.01; *** - p<0.001.

Our data suggesting that sub-lethal knockdown of PTPMT1 can sensitize cells to sub-lethal doses of chemotherapeutic suggested that sub-lethal doses of alexidine dihydrochloride could also resensitize cells to chemotherapeutic. Using the dose response curves analyzed in Figure 6, we treated HeLa cells with a sub-lethal dose of either 0.5 µM or 1 µM of alexidine dihydrochloride for 24 hours. Importantly, neither concentration of drug induced cell death in this time frame (Figure 7B-D). We then exposed the alexidine dihydrochloride treated cells, or cells treated with a vehicle only control, to a dose response of paclitaxel for an additional 24 hours before assaying viability. Both doses of alexidine dihydrochloride were sufficient to sensitize cells to a wide range of paclitaxel concentrations. Interestingly, 0.5 µM alexidine dihydrochloride was not able to significantly sensitize cells to doses of paclitaxel below its EC_50_ (doses ranging from 0.01 to 7.8 nM, Figure 8A). However, at higher doses, all of which exceed 15.6 nM paclitaxel, alexidine dihydrochloride pre-treatment is able to significantly sensitize cells to paclitaxel treatment (for 15.6 nM dose, p<0.05, for 31.25–250 nM paclitaxel, p<0.01, Figure 8A). In comparison, 1 µM alexidine dihydrochloride sensitizes cells to paclitaxel at all doses examined (for 0.01 nM dose, p<0.05; for all other doses, p<0.001, Figure 8B). To confirm these data, we determined the percentage of cells exposed to sublethal levels of alexidine dihydrochloride (0.5 µM or 1 µM) that stain positively for propidium iodide after treatment with 1 nM paclitaxel. While treatment of cells with a vehicle only has minimal effects to control cells exposed to paclitaxel (8.0% v. 8.6% cell death, p = 0.24, n.s., Figure 8C), pre-treatment of cells with either 0.5 µM or 1 µM alexidine dihydrochloride before treatment with paclitaxel significant increases the cell population undergoing cell death (For 0.5 µM AD treatment, 6.3% v. 14.2% cell death, p<0.001; for 1 µM AD treatment, 7.7% v. 20.5% cell death, p<0.05, Figure 8C). Collectively, these data demonstrate that treatment of HeLa cells with alexidine dihydrochloride is sufficient to sensitize cells to paclitaxel, and suggests that inhibition of PTPMT1, through siRNA-mediated knockdown or through alexidine dihydrochloride treatment, is sufficient to sensitize cells to paclitaxel.

Upon seeing a synergistic interaction between alexidine dihydrochloride and paclitaxel, similar to what was seen with PTPMT1 RNAi-mediated knockdown, we sought to determine if alexidine dihydrochloride was selectively targeting PTPMT1 in cells as we had seen in our *in vitro* assays. We hypothesized that if PTPMT1 is the main pharmacological target of alexidine dihydrochloride in cells, PTPMT1 knockdown cells should be significantly less sensitive to this drug. To determine this, we knocked down PTPMT1 using two independent siRNAs in cells for 30 hours before exposing these cells to a dose response curve of alexidine dihydrochloride for 24 hours. Importantly, no toxicity due to PTPMT1-mediated gene knockdown is seen within 54 hours of knockdown (Figure 1F), allowing us to confirm that the cell death in the assay was solely due to alexidine dihydrochloride toxicity. Figures 8D and E demonstrate that the knockdown of PTPMT1 did not significantly affect the response of HeLa cells to alexidine dihydrochloride across a large range of doses. We had previously shown that PTPMT1 knockdown affects cellular ATP levels, causing them to increase in glucose-containing medium, and hypothesized that treatment of HeLa cells with alexidine dihydrochloride could promote a similar metabolic response. Interestingly, while treatment of cells with 0.5 µM alexidine dihydrochloride did not significantly alter cellular ATP levels (Figure 8F, p = 0.25, n.s.), treatment with 1 µM alexidine dihydrochloride significantly decreased cellular ATP levels to only 52% of levels seen in untreated cells (Figure 8F, p<0.001). Overall, these data suggest that while alexidine dihydrochloride may be a good candidate drug for sensitizing cancer cells to chemotherapeutic treatment, that these effects are largely independent of PTPMT1 activity *in vivo*.

## Discussion

The mitochondrial phosphatase PTPMT1 has previously been implicated in affecting various aspects of mitochondrial biology, including ATP production, respiratory status, mitochondrial morphology, and the mitochondrial phosphoproteome [7], [9], [22]. Interestingly, however, no current reports link the direct ablation of PTPMT1 activity to the induction of mitochondrial-dependent apoptosis.

A total mouse knockout of PTPMT1 results in embryonic lethality, demonstrating its necessity in early embryonic development [9], [22]. Mouse embryonic fibroblasts (MEFs) derived from these mice and treated with Cre recombinase *ex vivo* were noted to have severe defects in mitochondrial morphology, metabolism, and cardiolipin production [9]. Additionally, PTPMT1 knockdown has previously been characterized in an insulinoma cell line, in which transient knockdown of this gene was sufficient to induce significant increases in cellular ATP levels relative to a non-targeting siRNA control [7]. In these studies, however, the downregulation of PTPMT1 expression levels did not dramatically influence cellular viability. These data suggest that the ablation of PTPMT1 function is not sufficient to induce apoptosis in all cell types, and that its loss in the cancer cells investigated here demonstrate a unique and distinct phenotype.

Although PTPMT1 loss of function has not previously been implicated in apoptosis, dysregulation of cardiolipin, as well as its enzymatic effectors, has been extensively documented in the context of this cellular process [12], [14], [17], [20]. The role of cardiolipin in apoptosis is twofold: cardiolipin influences both the docking of pro-apoptotic proteins to the mitochondrial outer membrane as well as the ability of cytochrome c to be released upon membrane permeabilization. Cardiolipin synthase (also known as CLS1, or gene name *CRLS1*) is critical for both the synthesis and maintenance of cellular cardiolipin levels. A recent study demonstrated that stable RNAi-mediated knockdown of this enzyme sensitized cells to apoptotic cell death ligands such as TNF-α [14]. This is consistent with our data demonstrating that siRNA knockdown of PTPMT1 also decreases total cardiolipin levels and sensitizes cells to apoptotic insults such as chemotherapeutic challenge. Collectively, these data suggest that therapies targeting the cardiolipin biosynthetic pathway may be a novel treatment paradigm for cancer patients, and may benefit their therapeutic regimens by enhancing chemotherapeutic efficiency and apoptotic response.

Interestingly, the authors of the CLS1 knockdown study noted that there were no significant viability defects observed in HeLa cells stably expressing shRNAs toward CLS1, despite significant reductions in cardiolipin levels (to approximately 25% relative to wild type HeLa cells). This suggests that a substantial decrease in cardiolipin levels is not sufficient to induce an apoptotic program. Similar results have been seen in yeast models; CLS1-null yeast, which have no detectable cardiolipin levels, are viable and grow equally well on fermentable and non-fermentable carbon sources [23]. These data demonstrate that cardiolipin biosynthesis is not required for viability in at least a subset of model systems. Given the apoptotic induction seen in cancer cells when PTPMT1 is knocked down, it is possible that this gene has additional functions which are critical for cancer cell viability yet independent of cardiolipin synthesis. PTPMT1 could have additional lipid or protein substrates under its regulation, and dysregulation of phosphorylation events on these substrates could lead to apoptosis irrespective of cardiolipin levels. Indeed, RNAi-mediated knockdown of PTPMT1 has been shown to induce a wide array of changes in the phosphorylation of unidentified mitochondrial proteins in pancreatic beta cells [7]. The dysregulation of these phosphorylation events, whether direct or indirect, could lead to changes in cell death. Additionally, PTPMT1 was recently suggested to regulate the phosphorylation of PI(3,5)P_2_, a phosphoinositide found at low levels in cells [22]. The authors of this study indicate that PI(3,5)P_2_ can localize to the mitochondria, suggesting a possible role of PTPMT1 in the stability, synthesis, or turnover of this lipid. Importantly, the cellular consequences of dysregulated mitochondrial PI(3,5)P_2_ are unknown.

Interestingly, previous work has demonstrated that ablation of PTPMT1 gene expression in MEFs or embryonic stem (ES) cells induces similar metabolic phenotypes seen in our experiments with PTPMT1 knockdown cells (20, 27). We report that PTPMT1 knockdown cells have significantly increased ATP/cell levels when cultured in glucose-containing media, but do not demonstrate elevated ATP levels when grown in pyruvate-containing media, which forces cells to bypass the glycolytic program (Figure 6C). These data suggest that the PTPMT1 knockdown-dependent increase in ATP levels specifically relies on glucose metabolism, and suggests that these cells may be upregulating glycolysis. These data are substantiated by metabolic observations in PTPMT1^−/−^ MEFs and ES cells, which both demonstrate a statistically signficant increased extracellular acidification rate (ECAR) relative to their wild type counterparts (20, 27). These data suggest that downregulation of PTPMT1 function in a variety of cell types causes a shift to glycolysis, perhaps to compensate for general deficiencies in mitochondrial metabolism that are associated with cardiolipin dysregulaion.

The increase in total ATP levels is a particularly intriguing phenotype, as, in our cell model system, it precedes significant apoptosis in PTPMT1 knockdown cells. As previously mentioned, PTPMT1 downregulation seems to result in an upregulation or shift to a glycolytic metabolic program in multiple cell types. Our data demonstrates elevated ATP levels 48 hours post-siRNA transfection, a time point at which the cells are still viable (Figure 1F). It is reasonable to believe that this upregulation in ATP levels, via glycolysis, could be a transient compensatory phase in which the cells are utilizing a non-mitochondrial pathway to generate energy. Subsequently, we hypothesize that this temporary metabolic shift cannot be sustained long-term in cancer cells, and, 72–96 hours after silencing of PTPMT1, leads to cancer cell death by apoptosis.

It is interesting to note that while whole-organism knockout of PTPMT1 is embryonic lethal (20, 27), ablation of PTPMT1 gene expression in non-transformed cells, including fibroblasts and ES cells, does not cause overall toxicity (20, 27). This is particularly curious due to the fact that the metabolic alterations we see in PTPMT1 knockdown cells, including diminished cardiolipin levels and a switch to a glycolyic metabolic program, are also seen in these cell types. We believe that this difference in cell fate is due to inherent metabolic differences seen in primary, non-transformed cells and cells derived from tumors. It is possible that the alterations in metabolism seen in PTPMT1-null cells are tolerated in normal, non-transformed cells, but are not compatible with viability it a transformed cellular state. It is well known that mitochondrial function, especially at the level of metabolism, is altered in cancer cells relative to normal cells. It is possible that some aspect of cellular transformation increases a cell’s dependency on PTPMT1, intact cardiolipin biosynthesis, or overall mitochondrial health for survival. Understanding the role that PTPMT1 plays in these distinct cellular contexts will be important to ultimately elucidating its cellular function.

Alexidine dihydrochloride was previously identified as a selective inhibitor of PTPMT1 through an *in vitro* screening approach [15], allowing the possibility of specific targeting of this phosphatase in cellular model systems. While our studies suggest that alexidine dihydrochloride truly is selective for PTPMT1 relative to other phosphatases *in vitro*, we also found that there is no significant response rate for HeLa cells transfected with a non-targeting or PTPMT1-specific siRNA. Interestingly, alexidine dihydrochloride treatment shares many intriguing phenotypes with PTPMT1 knockdown: the compound has previously been noted for its ability to induce mitochondrial dysfunction and subsequent apoptosis in a variety of cancer cell lines [21]. Interestingly, however, our studies also identify separate, independent phenotypes of PTPMT1 knockdown and alexidine dihydrochloride treatment; while PTPMT1 knockdown increases total levels of ATP/cell, alexidine dihydrochloride treatment causes significant decreases in cellular ATP levels at higher concentrations. Collectively, these data suggest that while alexidine dihydrochloride may be a novel therapeutic for sensitizing tumor cells to chemotherapy, this sensitization is probably functions largely independent of PTPMT1 expression in cells.

Overall, our study demonstrates that downregulation of PTPMT1 is sufficient to induce apoptosis at high doses, or sensitizes cancer cells to chemotherapeutic treatment at low doses. Though our data suggests that alexidine dihydrochloride is not selective for PTPMT1 in cells, this study justifies the need for further discovery and development of a PTPMT1 inhibitory compound for use as an adjuvant therapeutic strategy in tumors. Importantly, current data implies that inhibition of PTPMT1 in normal, non-transformed cells does not cause cell death and thus, within a critical therapeutic window, could be a specific treatment for tumor cells while minimizing off-target effects to normal cells.

## Materials and Methods

### Cell Culture and Reagents

HeLa cells (ATCC) were cultured in DMEM with a final concentration of 10% FBS. U2OS cells (ATCC) were cultured in McCoy’s media supplemented with 10% fetal bovine serum (FBS). H157 cells [24], as well as H1299, 786-0 and ACHN cells [25] were cultured in RPMI with 10% FBS. HOS cells (ATCC) were cultured in MEM-α supplemented with 10% FBS. Control and PTPMT1 siRNAs (Qiagen) or Bax and Bak1 pooled siRNAs (Dharmacon) were transfected into cells with Oligofectamine (Invitrogen). Knockdown efficiency was assayed using RT-PCR as previously described [26]. For western blotting of PTPMT1, HeLa cells were transfected with each PTPMT1 siRNA for 48 hours. For overexpression experiments, 500 ng FLAG-PTPMT1 expressed in pRK7 was overexpressed for 20 hours (48 hr total siRNA transfection time). Cell lysates were generated and probed as previously described [27]. Antibodies used include anti-FLAG (Sigma-Aldrich), anti-PTPMT1 (a gift from Dr. David Pagliarini, characterized in [7]), and anti-tubulin (Sigma-Aldrich). Paclitaxel (Sigma-Aldrich) and alexidine dihydrochloride (Tocris Bioscience) were resuspended in DMSO according to manufacturer’s directions.

### Cell Viability Assays

Cell Titer Glo (Promega) was used to detect cellular viability according to the manufacturer’s directions. Real-time cell viability was determined using the xCELLigence system, as previously described [27]. Propidium iodide (PI) positivity was determined and analyzed as previously described [27]. APC-conjugated Annexin V (Invitrogen) was used to detect apoptosis in live cells according to manufacturer’s instructions. Antibodies toward cleaved caspase-3 (Cell Signaling #9661) and cleaved PARP (Cell Signaling #9541) were used to determine apoptosis in cells transfected with PTPMT1 siRNAs. Cells were collected, washed, and fixed in 1% formaldehyde for 10 min. After 3 PBS washes, 100% ice cold methanol was added to cells, which were allowed to permeabilize at −20°C overnight. Cells were washed, blocked in 0.5% BSA for 30 minutes (room temperature) and exposed to each primary antibody (1∶1000 Cl. Casp-3 or 1∶25 Cl. PARP) for 1 hr. AF488-conjugated anti-rabbit secondary antibody was added (1∶1000) and incubated for 30 min. Cleaved caspase-3, and cleaved PARP positivity were determined using a FACSCaliber flow cytometer using the FL1 channel to determine fluorescence, with at least 10,000 events captured per experimental condition; APC-Annexin V was determined on the FL4 channel under the same conditions. Data was analyzed on CellQuest software. For analysis of fold change in cell death, the percentage of cells transfected with a non-targeting control was set as 1; increases in cell death beyond this percentage are depicted as increased fold change in cell death. The heatmap was generated using Gene-E (Broad Institute).

### Cardiolipin and ATP Determination Assays

To determine cardiolipin levels, HeLa cells were radiolabeled with ^32^P-orthophosphate as previously described [28]. Briefly, 72 hours of transfection, normal growth medium was replaced with phosphate-free DMEM (Invitrogen) supplemented with 10% phosphate-free FBS for 1 hour. 0.1 mCi of [^32^P]O4 (Perkin Elmer) was added to each sample and incubated for an additional 4 hours. Radiolabeling was stopped with TCA (10% final concentration). Cells were scraped, pelleted and lipids extracted via an acidified Bligh and Dyer method. Lipids were lyophilized, resuspended in chloroform:methanol (1∶1), spotted on silica gel TLC plates (Whatman) and cardiolipin was resolved in a buffer containing chloroform, methanol, and acetic acid (65∶28∶8), as previously described [14]. The TLC plate was exposed to film for 20 hours at −80°C and developed. Lipids were quantified using the phosphorimager according to manufacturer’s instructions. To determine cellular ATP/cell, cellular ATP levels were determined using Cell Titer Glo (Promega) according to manufacturer’s instructions. These levels were normalized to total cell number, as assayed via CyQuant (Invitrogen) per manufacturer’s instructions.

### Preparation of Recombinant Proteins and Phosphatase Assays

The human PTPMT1 open reading frame was cloned into pGEX-KG, sequence verified, and transformed into BL21 competent cells (Sigma). Protein expression was induced using isopropyl β-D-1-thiogalactopyranoside (IPTG) and subsequent incubation at 23°C with constant rotation overnight. MKP-3 was cloned into pDEST15 (Invitrogen), sequence verified, and transformed into BL21-AI bacteria (Invitrogen). Expression was induced using a final concentration of 0.2% L-arabinose (w/v) and inducing at 27°C with constant rotation for 4 hours. Proteins were purified using glutathione-conjugated sepharose and stored at −80°C until use. Phosphatase assays were performed using ∼100 ng of each recombinant protein, which was spiked into phosphatase assay buffer (50 mM sodium acetate, 25 mM Tris-HCl, 1 mM DTT, pH 6.0 for PTPMT1, pH 7.0 for MKP-3) and 100 mM O-methylfluoroscien-phosphate (OMFP, Sigma-Aldrich). Alexidine dihydrochloride was titrated from 1 mM to 1 nM concentrations and added to each reaction, in addition to a vehicle only control. Reactions were incubated at 37°C for 1 hr, and fluorescence was measured as a readout of phosphatase activity.
